# Filaggrin haploinsufficiency is highly penetrant and is associated with increased severity of eczema: further delineation of the skin phenotype in a prospective epidemiological study of 792 school children

**DOI:** 10.1111/j.1365-2133.2009.09339.x

**Published:** 2009-10

**Authors:** SJ Brown, CL Relton, H Liao, Y Zhao, A Sandilands, WHI McLean, HJ Cordell, NJ Reynolds

**Affiliations:** * Department of Dermatology, Royal Victoria InfirmaryNewcastle upon Tyne NE1 3LP, U.K.; † Institute of Human Genetics, Newcastle UniversityNewcastle upon Tyne, U.K.; § Institute of Cellular Medicine, Newcastle UniversityNewcastle upon Tyne, U.K.; ‡ Epithelial Genetics Group, Division of Molecular Medicine, Colleges of Life Sciences and Medicine, Dentistry and Nursing, University of DundeeDundee, U.K.

**Keywords:** atopic, eczema, filaggrin, ichthyosis vulgaris, phenotype

## Abstract

**Background:**

Null mutations within the filaggrin gene (*FLG*) cause ichthyosis vulgaris and are associated with atopic eczema. However, the dermatological features of filaggrin haploinsufficiency have not been clearly defined.

**Objectives:**

This study investigated the genotype–phenotype association between detailed skin phenotype and *FLG* genotype data in a population-based cohort of children.

**Methods:**

Children (*n*= 792) aged 7–9 years were examined by a dermatologist. Features of ichthyosis vulgaris, atopic eczema and xerosis were recorded and eczema severity graded using the Three Item Severity score. Each child was genotyped for the six most prevalent *FLG* null mutations (R501X, 2282del4, R2447X, S3247X, 3702delG, 3673delC). Fisher’s exact test was used to compare genotype frequencies in phenotype groups; logistic regression analysis was used to estimate odds ratios and penetrance of the *FLG* null genotype and a permutation test performed to investigate eczema severity in different genotype groups.

**Results:**

Ten children in this cohort had ichthyosis vulgaris, of whom five had mild–moderate eczema. The penetrance of *FLG* null mutations with respect to flexural eczema was 55·6% in individuals with two mutations, 16·3% in individuals with one mutation and 14·2% in wild-type individuals. Summating skin features known to be associated with *FLG* null mutations (ichthyosis, keratosis pilaris, palmar hyperlinearity and flexural eczema) showed a penetrance of 100% in children with two *FLG* mutations, 87·8% in children with one *FLG* mutation and 46·5% in wild-type individuals (*P*< 0·0001, Fisher exact test). *FLG* null mutations were associated with more severe eczema (*P*= 0·0042) but the mean difference was only 1–2 points in severity score. Three distinct patterns of palmar hyperlinearity were observed and these are reported for the first time.

**Conclusions:**

Filaggrin haploinsufficiency appears to be highly penetrant when all relevant skin features are included in the analysis. *FLG* null mutations are associated with more severe eczema, but the effect size is small in a population setting.

Filaggrin (‘*fil*ament *aggr*egating prote*in*’) is a multifunctional protein expressed by terminally differentiating keratinocytes. It aggregates and aligns keratin intermediate filaments, to facilitate the collapse of granular layer keratinocytes to produce squames.^1^ Filaggrin is subsequently degraded to release a mixture of amino acids: these contribute to the natural moisturizing factor in the stratum corneum;^2^ they also reduce the skin surface pH^3^ and provide histidine as a putative cutaneous ultraviolet photoreceptor.^4^ The molecular structure of filaggrin includes an S100 calcium-binding domain and it may therefore play an additional role in calcium signalling.^5^

Loss-of-function (null) mutations within the gene encoding filaggrin (*FLG*) cause ichthyosis vulgaris^6^ and are significantly associated with atopic eczema.^7^ The strong and significant association of *FLG* null mutations with atopic eczema has been demonstrated and replicated in multiple case–control, family- and population-based studies.^8^,^9^ The strongest and most highly significant associations have been reported with early-onset, persistent eczema^10^,^11^ (odds ratio 5·6, 95% confidence interval 4·1–7·8, *P*= 1·3 × 10^−28^) and asthma occurring in individuals with atopic eczema^12^–^14^ (odds ratio 3·49, 95% confidence interval 2·00–6·08, *P*= 1·0 × 10^−5^). Genetic epidemiological studies have shown that *FLG* null mutations are significantly associated with palmar hyperlinearity,^15^–^17^ keratosis pilaris,^17^ fine scaling^16^,^17^ and self-reported ‘dry skin’,^16^ each of which may be features of ichthyosis vulgaris. However, the clinical skin phenotype resulting from filaggrin haploinsufficiency (meaning a deficiency of filaggrin as a result of loss-of-function mutations) has not been clearly defined.

Further analysis of a carefully phenotyped cohort of children for whom detailed *FLG* genotype information is available provides a useful tool with which to investigate the genotype–skin phenotype association.

## Materials and methods

### Skin examination

English school children (*n*= 792) aged between 7 and 9 years from an unselected population birth cohort (the North Cumbria Community Genetics Project) were examined, each on one occasion, by an experienced dermatologist (S.J.B.), as part of a larger genetic epidemiological study, reported previously.^17^ The study was approved by the Local Research Ethics Committee and a parent or guardian of each child gave written informed consent.

Each child’s skin was examined for eczema, features associated with atopic eczema, ichthyosis and xerosis. Ichthyosis vulgaris was defined as fine scaling on the extensor surfaces of the limbs in addition to keratosis pilaris and palmar hyperlinearity. Xerosis was defined as skin that was dry to the touch, but without visible scale, hence the categories of ichthyosis and xerosis are mutually exclusive. Flexural eczema on examination was used as the case definition for this analysis in order to focus on cases of atopic eczema. Eczema severity was scored using the Three Item Severity (TIS) score,^18^ a simple, validated scale that allows the rapid assessment and recording of clinically significant features: 1–2 represents mild eczema, 3–5 represents moderate eczema and 6–9 represents severe eczema.^19^

For the purposes of this report, we have used the nomenclature recommended by the review committee of the World Allergy Organization,^20^ in which ‘eczema’ is used as an umbrella term to encompass ‘atopic eczema’ and ‘nonatopic eczema’.

### Genetic analysis

A DNA sample from each child was analysed for the six most prevalent *FLG* null mutations in the European population (R501X, 2282del4, R2447X, S3247X, 3702delG and 3673delC) using previously reported methods.^21^,^22^

### Statistical analysis

Genotype frequencies in different phenotype groups were compared by Fisher’s exact test, under the null hypothesis that there is no association with genotype. Logistic regression analysis was used to estimate the odds ratio and penetrance of the *FLG* null genotype. A permutation test was performed to compare eczema severity scores in the three different genotype groups (wild-type homozygotes, heterozygotes and individuals with two *FLG* null mutations, either homozygous mutants or compound heterozygotes). Analyses were carried out using the statistical analysis package Stata (version 9, Stata for Linux; StataCorp LP, College Station, TX, U.S.A.).

The six screened *FLG* null mutations were considered to be a single null allele for the purposes of this analysis; the rationale for this approach is based on biochemical and immunohistochemical evidence that each null mutation produces truncated forms of profilaggrin which result in a marked reduction or absence of processed filaggrin when present in the homozygote or compound heterozygote state.^6^,^21^

## Results

The demographic data and clinical features of the 792 children are shown in Table 1. Ten children had classical ichthyosis vulgaris and, among these, five had flexural eczema (point prevalence) and three had nonflexural as well as flexural eczema. No child had ichthyosis vulgaris and exclusively nonflexural eczema. Two of the children with ichthyosis vulgaris had mild eczema and three had eczema of moderate severity.

**Table 1:** Demographic data and clinical features of the skin for 792 English school children aged 7–9 years

Demographic data and clinical features	Number (%)
Sex	
Male	405 (51·1)
Female	387 (48·9)
Flexural eczema	120 (15·2)
Ichthyosis vulgaris	10 (1·3)
Milder ichthyosis	54 (6·8)
Xerosis	193 (24·4)
Keratosis pilaris	273 (34·4)
Palmar hyperlinearity	167 (21·1)

Clinical features are recorded on the basis of skin examination by a single experienced dermatologist (S.J.B.) and represent point prevalence. ‘Ichthyosis vulgaris’ is defined as scaly skin on extensor surfaces plus hyperlinear palms and keratosis pilaris; ‘milder ichthyosis’ is defined as scaly skin as an isolated feature; ‘xerosis’ is defined as clinically dry skin without scaling. All skin examinations were performed during the winter months.

As reported previously, *FLG* null mutations are significantly associated with the predominantly mild–moderate eczema seen in this cohort.^17^ Our study utilized an unselected population cohort and hence the true risk or penetrance of *FLG* null mutations can be assessed, i.e. the proportion of individuals with a given genotype having a given phenotype.

These data demonstrate that the penetrance of *FLG* null mutations with respect to flexural eczema in this cohort is 55·6% in homozygotes and 16·3% in heterozygotes, compared with 14·2% in wild-type homozygotes. The penetrance with respect to skin signs known to be associated with *FLG* haploinsufficiency^15^–^17^ (summing ichthyosis, keratosis pilaris, palmar hyperlinearity and flexural eczema) is 100% in homozygotes, 87·8% in heterozygotes and 46·5% in wild-type homozygotes (*P*< 0·0001, Fisher exact test). Xerosis is not included in this grouped analysis as xerosis as a skin sign (meaning clinically dry skin in the absence of scaling) is not independently associated with *FLG* null mutations.^17^ The equivalent odds ratios for *FLG*-related skin features are > 100 in *FLG* null homozygotes and 6·3 in *FLG* heterozygotes, compared with 1·0 in wild-type individuals. Furthermore, in this cohort the association of *FLG* null mutations with the combined ichthyosis and/or keratosis pilaris and/or hyperlinear palms and/or eczema group persists when the 10 cases of ichthyosis vulgaris are excluded from analysis (*P*< 0·0001).

Of the 778 children for whom *FLG* genotype data were available for all six of the screened mutations, there was one homozygote, eight compound heterozygotes (i.e. children carrying two different *FLG* null alleles) and 98 heterozygotes. None of the nine children with two *FLG* mutations had entirely normal skin: five had eczema, seven had classical ichthyosis vulgaris (including hyperlinear palms and keratosis pilaris) and the remaining two had milder ichthyosis. Skin signs observed in the 98 heterozygous children are summarized in Table 2. Only 10 of the 98 heterozygotes (10·2%) had clinically normal skin, whereas 281 of the 671 (41·9%) homozygous wild-type individuals had normal skin, a statistically significant difference (*P*< 0·0001).

**Table 2:** Skin phenotypes and clinical features in 778 children with different filaggrin null genotypes

Skin phenotype or clinical feature	*FLG* null homozygotes^a^ (*n* = 9), *n* (%)	*FLG* null heterozygotes (*n* = 98), *n* (%)	*FLG* wild-type homozygotes (*n*= 671), *n* (%)
Flexural eczema	5 (55·6)	16 (16·3)	95 (14·2)
Ichthyosis vulgaris	9 (100)	3 (3·1)	0 (0)
Milder ichthyosis	2 (22·2)	23 (23·5)	30 (4·5)
Xerosis	0 (0)	32 (32·7)	163 (24·3)
Keratosis pilaris	9 (100)	59 (60·2)	201 (30·0)
Hyperlinear palms	9 (100)	73 (74·5)	84 (12·5)
Clinically normal skin	0 (0)	10 (10·2)	281 (41·9)

Genotyping results for all six of the screened mutations (R501X, 2282del4, R2447X, S3247X, 3702delG, 3673delC) were available for these 778 children out of the 792 children included in this cohort study. Analysis is performed using a combined null genotype, in which the different *FLG* null mutations are considered to be equivalent, on the basis of their equivalent effects demonstrated in previous biochemical and immunohistochemical studies.^21^^a^The group of *FLG* null homozygotes includes one R501X homozygote and eight compound heterozygotes.

**Fig 1:**
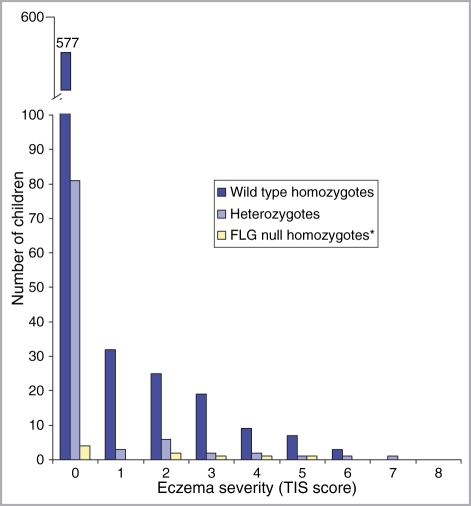
Column chart to illustrate the eczema severity scores of 778 children with different filaggrin genotypes. TIS score, Three Item Severity score,^18^ where a score of 1–2 represents mild eczema, 3–5 moderate and 6–9 severe disease.^19^*Individuals with two *FLG* null mutations include one homozygote (with two copies of the R501X null mutation) and seven compound heterozygotes (with two different null mutations). A permutation test demonstrates that there is a significant difference in eczema severity when the three genotype groups are compared (*P*= 0·0042).

The column chart in Figure 1 shows the eczema severity scores in children with flexural eczema having different *FLG* genotypes. The chart illustrates that children with one or more *FLG* null mutation tended to have more severe eczema: a permutation test comparing the three genotype groups – wild-type homozygotes, heterozygotes and *FLG* null homozygotes – shows that this difference is statistically significant (*P*= 0·0042, comparing all three groups).

**Fig 2:**
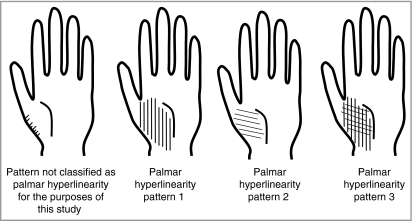
Diagrammatic representation of the patterns of palmar skin markings observed in a series of 308 English school children aged 7–9 years. The palmar skin features of a total of 792 children were examined by a single experienced dermatologist (S.J.B.). Examination of 484 children led to the observation of three distinct patterns of palmar hyperlinearity and the patterns of palmar markings were then recorded for the subsequent 308 children. The majority of children in this cohort had very smooth palmar skin; within the series of 308 children, 65 had palmar hyperlinearity of whom 18% showed pattern 1, 20% showed pattern 2 and 62% showed pattern 3. Diagrams have been used to illustrate the hyperlinearity patterns as clinical photos were not permitted because of ethical constraints, but examples of the distinct patterns may be seen in published literature.^6^,^22^,^36^–^39^

Examination of such a large number of children led to the novel observation that there are several distinct patterns of palmar hyperlinearity, illustrated diagrammatically in Figure 2. The vast majority of children in this cohort had smooth palmar skin; some children had a subtle increase in palmar markings at the margins of the thenar eminence, but this was not classified as palmar hyperlinearity for the purposes of this study. The three distinct patterns of palmar hyperlinearity were defined after examination of 484 children; in the subsequent series of 308 children, 65 cases of palmar hyperlinearity were observed. Within this series, the majority of children (40 of 65; 62%) showed pattern 3, and roughly equal numbers showed pattern 1 (12 of 65; 18%) and pattern 2 (13 of 65; 20%). Pattern 1 predominated in the children with ichthyosis vulgaris (seen in four of five children with ichthyosis vulgaris). Pattern 3 was most common in children with eczema (seen in 10 of 21 eczema cases) but it was also frequently observed in children with otherwise normal skin (29 of 39 children with normal skin in this series).

## Discussion

The causal association of *FLG* null mutations with ichthyosis vulgaris has previously been demonstrated by pedigree examination, histological and biochemical data,^6^ and a strongly significant association with atopic eczema has been established using genetic epidemiology.^9^,^23^ However, this study furthers our understanding of the skin phenotype associated with filaggrin haploinsufficiency in two main ways: firstly, the strong penetrance of eczema, ichthyosis and associated skin signs in different *FLG* genotypes is demonstrated in an unselected population; and secondly, the influence of *FLG* null mutations on eczema severity is assessed.

Use of an unselected population-based cohort allows estimation of the true risk or penetrance of *FLG* null mutations, rather than the relative risk which can be estimated from case–control studies or atopy-related cohorts. The penetrance of *FLG* null mutations with respect to flexural eczema as a point prevalence is 55·6% in homozygotes and compound heterozygotes and 16·3% in heterozygotes, but the penetrance in heterozygotes is not significantly different from wild-type individuals, at 14·2%. The use of point prevalence of eczema as a case definition would almost certainly have led to an underestimation of the eczema penetrance in each genotype group, because of the relapsing and remitting nature of the disease. In contrast, the point prevalence of the more stable clinical signs such as keratosis pilaris and palmar hyperlinearity is more representative of the population period prevalence. Summation of ichthyosis, keratosis pilaris, palmar hyperlinearity and flexural eczema, to take into account all the skin features which may be associated with *FLG* null mutations, suggests a highly penetrant haploinsufficiency: 100% of individuals with two *FLG* null mutations and 87·8% of individuals with one *FLG* null mutation show one or more of these skin features, compared with 46·5% of wild-type individuals, a statistically significant difference (*P*< 0·0001, Fisher exact test). Furthermore, this significant difference persists when the 10 ichthyosis vulgaris cases are removed from the analysis, indicating that the effect is not purely driven by ichthyosis vulgaris. These observations are consistent with a semidominant pattern of inheritance in which the *FLG* null heterozygotes exhibit a milder skin phenotype than the *FLG* null homozygotes.^7^

The finding that 85–100% of individuals carrying one or more *FLG* null mutations may demonstrate signs of filaggrin haploinsufficiency is striking (notwithstanding a limitation to this study in that only the six most prevalent *FLG* null mutations were examined) although the clinical signs are often asymptomatic. However, incomplete penetrance (< 100%) implies that other factors – both genetic and environmental – modulate the effects of filaggrin haploinsufficiency, particularly in eczema where the penetrance is much lower than in the ichthyosis/keratosis pilaris/palmar hyperlinearity/eczema group used for the combined analysis. Experimental evidence for such genetic and environmental modulation includes the demonstration that cytokines from Th2 cells can downregulate filaggrin expression in atopic eczema skin;^24^ in addition, several different mouse models have demonstrated that mutations in other genes encoding stratum corneum proteins can affect filaggrin expression and/or processing.^25^–^29^

The cellular and molecular mechanisms by which filaggrin deficiency results in skin features such as palmar hyperlinearity and keratosis pilaris remain to be defined. It is noteworthy that approximately 46·5% of *FLG* wild-type individuals also demonstrated ichthyosis, keratosis pilaris, palmar hyperlinearity and/or eczema, which may be a result of filaggrin downregulation by other genetic and/or environmental factors or by filaggrin-independent mechanisms. The mechanisms by which filaggrin deficiency may result in ‘dry skin’ have been discussed.^30^,^31^ The fact that *FLG* haploinsufficiency is not independently associated with xerosis *per se* in this cohort^17^ may conflict with the hypothesis that *FLG* null mutations result in dry skin because of a dose reduction in hygroscopic amino acids,^30^,^31^ the so-called ‘natural moisturizing factor’.

To date only two studies have attempted to investigate whether eczema severity is associated with the presence or absence of *FLG* null mutations.^32^,^33^ This is a reflection of the fact that most *FLG*-related data have been generated from studies of moderate–severe cases, excluding children with mild disease. In contrast, our cohort study generates data relating predominantly to mild–moderate eczema cases, with very few (*n*= 5) severe cases, none of whom was an *FLG* null homozygote or compound heterozygote. Our analysis comparing severity scores between genotype groups does show a significant difference across all three groups (*P*= 0·0042, permutation test). This is in keeping with the analysis by Morar *et al.,* which showed a significant association between *FLG* genotype and eczema severity (*P*= 0·007 with the linear regression model).^32^ The only other reported analysis showed no significant association between the combined *SPINK5*, *KLK7* and *FLG* null genotype although the numbers were small (*n*= 99).^33^ However, the finding of a statistically significant association does not necessarily imply a clinically significant association. In the study cited above (Morar *et al.*) it was estimated that *FLG* had only a very minor effect on eczema severity (*R*^2^ = 0·8%).^32^ Similarly in our study, the mean eczema severity score is 1·8 in the group with two *FLG* null mutations compared with 0·4 in wild-type homozygotes, and the clinical relevance of a difference of 1–2 points on the TIS score is likely to be marginal. However, it has not been formally assessed in terms of quality of life or patient symptoms.

Keratosis pilaris and palmar hyperlinearity are each independently associated with *FLG* null mutations in this cohort.^17^ The association may be coincidental, as keratosis pilaris and hyperlinear palms are both features of ichthyosis vulgaris,^34^,^35^ but this is unlikely as the statistical association persists after excluding the ichthyosis vulgaris cases. The novel observation that there are three distinct patterns of palmar hyperlinearity merits further investigation: it may help to clarify the ultrastructural mechanisms by which filaggrin haploinsufficiency results in hyperlinearity, as the pattern of linear markings running perpendicular to the thenar eminence (pattern 1, Fig. 2) predominates in cases of ichthyosis vulgaris, although the cross-hatched pattern (pattern 3, Fig. 2) is more prevalent overall. These different patterns may be observed in previously published photographs: pattern 1 is shown by O’Regan and Irvine (Fig. 1b)^36^ and Judge *et al.*;^37^ pattern 2 is demonstrated by Nomura *et al.* (Figs 2c and 2d);^38^ while pattern 3 is demonstrated by Smith *et al.* (Figs 1c and 1e),^6^ Sandilands *et al.* (Fig. 1b)^22^ and Chen *et al.* (Figs 1a and 1b).^39^

In conclusion, filaggrin haploinsufficiency is a highly penetrant trait, associated with ichthyosis, keratosis pilaris, palmar hyperlinearity and flexural eczema, with a statistically significant trend towards more severe eczema. A greater understanding of the complex skin phenotype resulting from filaggrin deficiency may in the future facilitate the development and appropriate application of novel therapeutic strategies.
